# Relationship between novel anthropometric indices and the incidence of hypertension in Chinese individuals: a prospective cohort study based on the CHNS from 1993 to 2015

**DOI:** 10.1186/s12889-023-15208-7

**Published:** 2023-03-06

**Authors:** Xin Zhang, Runyu Ye, Lirong Sun, Xueting Liu, Si Wang, Qingtao Meng, Xiaoping Chen

**Affiliations:** 1 Cardiology Department, West China Hospital, Sichuan University, People’s Republic of China, 610041 Chengdu, Sichuan Province China; 2 Cardiology Department, West China Hospital, Sichuan University, 610041 Chengdu, Sichuan Province People’s Republic of China

**Keywords:** Hypertension, Incidence, A body roundness index, Body shape index

## Abstract

**Background:**

Recently, novel anthropometric indices (AHIs), including the body roundness index (BRI) and a body shape index (ABSI), were proposed to evaluate a subject’s nutritional status and metabolic disorders. In the present study, we mainly analyzed the relationship between AHIs and the incidence of hypertension and preliminarily compared their abilities to discriminate hypertension incidence in the Chinese population from the China Health and Nutrition Survey (CHNS).

**Methods:**

A total of 12,154 participants were included in this longitudinal study. The age range of this cohort was 18–94 years old (mean age: 40.73 ± 13.85 years old). 4511 participants developed hypertension during a median of 7.00 years of follow-up. Cox regression analysis, stratified analysis, and interaction tests were used to analyze the relationship between AHIs and the incidence of hypertension. Time-dependent receiver operating characteristic (ROC) curves, integrated discrimination improvement (IDI) and net reclassification index (NRI) were calculated to appraise the AHIs’ discrimination value of new-onset hypertension.

**Results:**

Kaplan‒Meier curves demonstrated that the participants in higher quartiles of AHIs (ABSI or BRI) at baseline were at greater risk of hypertension incidence during the follow-up. After adjusting for confounding factors, multivariate Cox regression models showed that the quartiles of BRI were significantly associated with an increased risk of hypertension in the whole cohort but were relatively weak for ABSI quartiles (*P* for trend = 0.387). In addition, ABSI z score (HR = 1.08, 95% CI: 1.04–1.11) and BRI z score (HR = 1.27, 95% CI: 1.23–1.30) were positively associated with increased incident hypertension in the total population. Stratified analysis and interaction tests showed a greater risk of new-onset hypertension in those < 40 years old (HR = 1.43, 95% CI: 1.35–1.50) for each z score increase in BRI and a higher incidence of hypertension in participants who were drinkers (HR = 1.10, 95% CI: 1.04–1.14) for each z score increase in ABSI. In addition, we observed that the area under the curve for identifying hypertension incidence for BRI was significantly higher than that for ABSI at 4, 7, 11, 12, and 15 years (all P < 0.05). However, the AUC of both indices decreased over time. Furthermore, the addition of BRI improved the differentiation and reclassification of traditional risk factors with a continuous NRI of 0.201 (95% CI: 0.169–0.228) and an IDI of 0.021 (95% CI: 0.015–0.028).

**Conclusion:**

Increased ABSI and BRI were associated with an increased risk of hypertension in Chinese individuals. BRI performed better than ABSI in identifying the new onset of hypertension, and the discrimination ability of both indices decreased over time.

## Background

Hypertension is one of the largest contributors to morbidity and mortality worldwide and is also the most important modifiable risk factor for cardiovascular disease [1]. According to the newest national hypertension survey, 23.2% of the population aged ≥ 18 years old has hypertension in China, accounting for 240 million patients [2]. It is estimated that high systolic pressure accounted for 2.54 million deaths in 2017 in China [3]. In addition, the direct medical costs related to diagnosis, tests, medication, outpatient visits, and hospitalization therapy for hypertension were 115.7 and 109.0 US dollars per patient per year in urban and rural areas of China [4]. Hence, hypertension causes a huge burden on people’s health and the social economy in our country. Positive prevention and identifying the high risk of hypertension incidence in the normotensive population have great significance.

Anthropometric indices (AHIs) are simple and accessible parameters to evaluate nutritional status. The most commonly used AHIs in clinical practice and epidemiological studies include body mass index (BMI), waist circumference (WC), hip circumference (HC), and waist-to-hip ratio (WHR). Prospective cohort studies have demonstrated that BMI and WHR could discriminate the incidence of hypertension [5–7]. This suggests that AHIs might help to identify subjects with high risk and to supply a precise prevention strategy for the specific population. Which could further reduce the burden caused by hypertension.

Recently, two novel AHIs have been developed, named the body shape index (ABSI) and body roundness index (BRI) [8, 9]. ABSI, proposed by Krakauer et al. in 2012, is based on WC, height, and BMI and can predict mortality independently from BMI in the United States population [8]. Meanwhile, Thomas et al. suggested BRI as a predictor of visceral adiposity tissue and body fat percentage [9]. BRI has proven to be a good predictor of metabolic syndrome in diverse nationalities and ethnic groups [10]. In recent years, some cross-sectional studies have compared the identification of hypertension by ABSI, BRI, and traditional AHIs in Chinese, Iranian, Peruvian, and European populations [11–14]. Generally, BRI was a significantly better tool to discriminate hypertension than ABSI [15]. To date, only one longitudinal study evaluated the hypertension discrimination value of novel AHIs in a Korean population [16]. Therefore, the relationship between novel AHIs and the incidence of hypertension in the Chinese population is unclear. In the present study, we mainly analyzed the relationship between AHIs and the incidence of hypertension and also preliminarily compared their abilities to discriminate hypertension incidence in the Chinese population from the China Health and Nutrition Survey (CHNS).

## Methods

### Study population

The longitudinal data of participants in the present study were from the CHNS. The CHNS is a nationwide survey on risk factors, nutrition, and health-related outcomes in the Chinese population from 15 provinces and autonomous districts, including Beijing, Chongqing, Guangxi, Guizhou, Heilongjiang, Henan, Hubei, Hunan, Jiangsu, Liaoning, Shaanxi, Shandong, Shanghai, Yunnan, and Zhejiang. The CHNS is an ongoing open cohort and an international collaborative project announced in 1989 and subsequently conducted in 1991, 1993, 1997, 2000, 2004, 2006, 2009, 2011, and 2015. In this project, a multistage, random cluster process was used to draw the samples surveyed in each of the provinces. Details of the cohort and sampling process have been published elsewhere [17].

To analyze the correlation between AHIs and the risk of hypertension, we used CHNS data from 1993 to 2015. Waist circumference (WC) and hip circumference (HC) were not collected in 1989 and 1991. Adults aged ≥ 18 years at the first wave with integrated data on sex, anthropometric indicators (including height, weight, WC, and WC), systolic blood pressure (SBP), diastolic blood pressure (DBP), ethnicity, and smoking and drinking status were suitable for this analysis. Participants with the following criteria were excluded: those with missing data for the abovementioned indices; those who had just one medical record over the years; those who were pregnant or lactating at the time of the survey; and those with extreme values (e.g., height < 120 cm; weight > 150 kg; WC < 50 cm or > 150 cm; and HC < 50 cm or > 150 cm). In addition, participants with hypertension at baseline (including those who had a self-reported diagnosis of hypertension, antihypertensive medication, and average SBP ≥ 140 mmHg or DBP ≥ 140 mmHg) were also excluded. Stata/SE version 15.1 was used for original data merging, calculation, cleaning and conversion before the statistical analysis, according to the inclusion and exclusion criteria as previously mentioned.

### Demographic parameters

Information on age, gender, race (Chinese Han or other), urban or rural residence, and current smoking and drinking status were gathered from the questionnaires at each follow-up survey. Current smoking was defined by whether participants themselves reported that they had still smoked cigarettes at each survey. Nonsmokers and former smokers were defined as noncurrent smokers. Drinking status was evaluated according to the frequency of alcohol consumption by self-report. In the present study, a participant who did not drink in the past year was defined as nondrinking, and the others (including drinking daily, 3–4 times/week, 1–2 times/week, and less than once a week) were defined as drinking status.

### Blood pressure measurements

The trained staff performed blood pressure measurements following the standard protocol and using appropriately sized cuffs at each follow-up survey. Before measurements, all participants were required to have a 10-min seated rest. Triplicate measurements of blood pressure on the right arm were conducted by using mercury sphygmomanometers, with at least 1 min between recordings [18]. The average of the three blood pressure measurements was calculated for the final analysis.

### Anthropometric measurements

The anthropometric measurements were administered by well-trained research staff in a private and comfortable room. All participants were requested to remove bulky clothing and shoes before measurement. Weight, height, WC, and HC were obtained using the calibrated equipment according to a standard procedure. ABSI was estimated as the WC divided by the BMI raised to two-thirds and by the square root of the height. In addition, the BRI was based on WC and height.

The specific formulas of ABSI and BRI are as follows [8, 9]:

$${\rm{ABSI}} = WC(m)*weight{(kg)^{ - 2/3}}*height{(m)^{5/6}}$$

$$\begin{array}{l}{\rm{BRI = 364}}{\rm{.2 - 365}}{\rm{.5}}\, \times \,{\rm{Eccentricity}}\,{\rm{Eccentricity}}\,{\rm{ = }}\\\,\,\,\,\,\,\,\,\,\,\,\,\,\,\,\sqrt {1 - \frac{1}{{{\pi ^2}}}{{\left( {{\raise0.7ex\hbox{${WC\left( m \right)}$} \!\mathord{\left/{\vphantom {{WC\left( m \right)} {Height\,(m)}}}\right.\kern-\nulldelimiterspace}\!\lower0.7ex\hbox{${Height\,(m)}$}}} \right)}^2}} \end{array}$$

### Study outcome

The outcome of the present study was new-onset hypertension during follow-up. Participants were identified if they had an average SBP ≥ 140 mmHg or DBP ≥ 90 mmHg, self-reported a diagnosis of hypertension, or were currently taking antihypertensive at any one of the follow-up visits [19, 20]. The first time for diagnosis with hypertension was considered the time when the end event occurred. For those free of hypertension in all follow-up surveys, the final survey date was used to calculate the follow-up time.

### Statistical methods

All statistical analyses were performed using R software (Version 4.2.1). Continuous data with a normal distribution were expressed as the mean ± standard deviation (SD). Categorical variables were expressed as the frequency. Continuous data were compared using the independent-samples t test. Differences in categorical variables were compared among the groups using the chi-squared test. The AHIs were converted into z scores and quartiles. In the etiological analysis section, Kaplan-Meier curves were used to evaluate the cumulative incidence for AHIs categories, and the log-rank test was utilized to examine the significance of the differences between groups. Univariate and multivariate Cox regression models were applied to analyze the association between each anthropometric measurement and the incidence of hypertension. The confounders in multivariate Cox regression were selected as those with significance in univariate analysis or reported by previous studies. Stratified analysis and interaction tests were conducted according to age (< 40 and > = 40 years old), sex, ethnicity (Chinese Hans and non-Hans), residence (urban and rural), current smoking status, current drinking status, SBP level (< 120 and > = 120 mmHg), and DBP level (< 80 and > = 80 mmHg). The hazard ratio (HR) of hypertension incidence and the 95% confidence intervals (95% CI) were calculated. In addition, we preliminarily analyzed the discrimination of hypertension incidence for ABSI and BRI, respectively. First, the ability to discriminate the incidence of hypertension was compared between ABSI and BRI using time-dependent receiver-operating characteristic (ROC) curve analysis. Second, owing to not collecting hypertension family history and serum biochemical indices in the early stage of CHNS, we could not directly use the existing prediction model in the literature. Furthermore, the integrated discrimination improvement (IDI) and net reclassification index (NRI) were calculated to appraise the incremental discrimination value of new-onset hypertension beyond the traditional factors based on age, sex, ethnicity, residence, smoking, drinking, SBP and DBP.

## Results

### Baseline characteristics

**Fig. 1:**
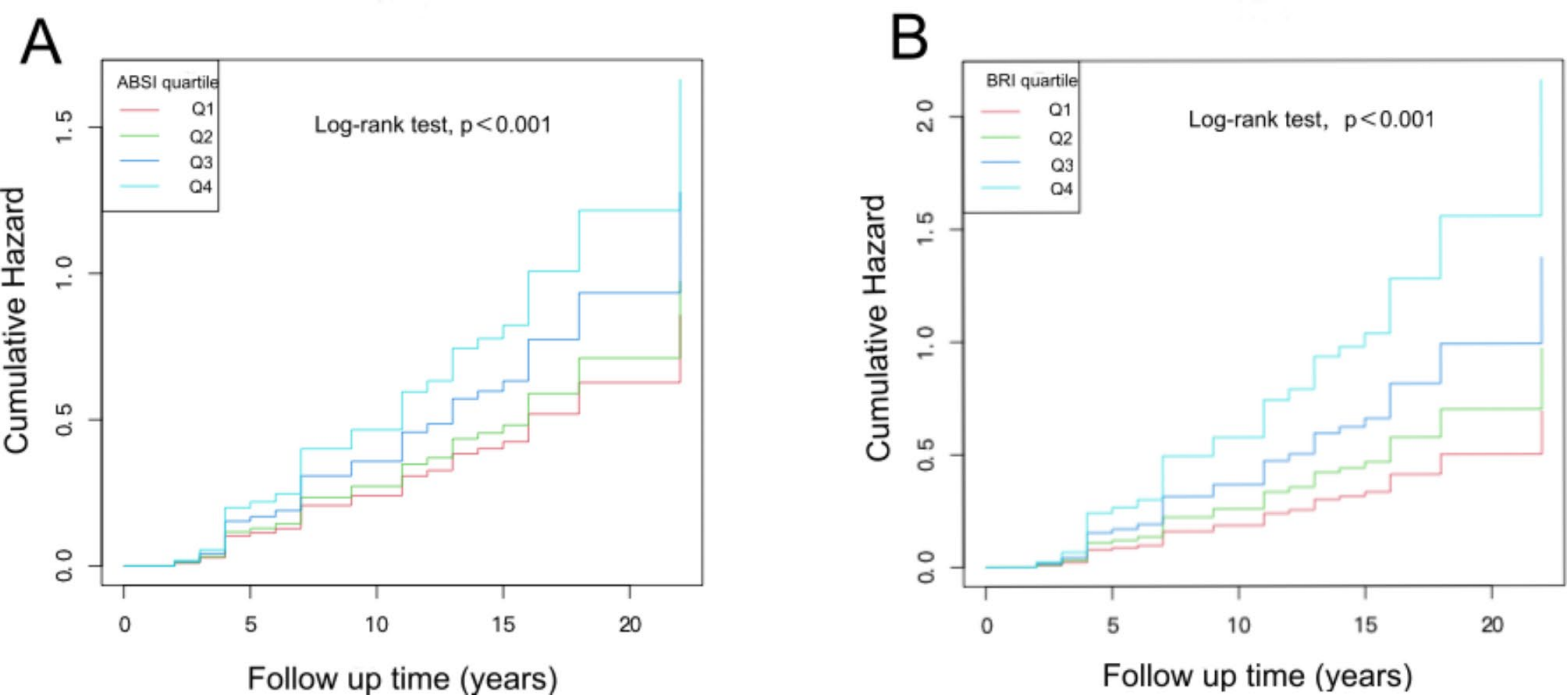
Kaplan-Meier curves of cumulative incidence of new- onset hypertension stratified by AHIs categories. A: for ABSI categories; B: for BRI categories

The age range of this cohort was 18–94 years old (mean age: 40.73 ± 13.85 years old). During a median of 7 years (1, 3 quartiles: 4, 15 years) of follow-up, 4511 participants (39.21/1000 person-years) developed hypertension, and 7463 were censored. Of them, 2291 incident cases of hypertension in 5484 men (43.49/1000 person-years) and 2220 cases in 6490 women (35.59/1000 person-years) were reported. The baseline characteristics of those who did and did not develop hypertension are presented in Table 1. There were significant differences between those who did develop hypertension and those who did not in the majority of the parameters except for height (*P* = 0.423). The participants who developed hypertension had higher age, weight, HC, WC, SBP, DBP, ABSI, and BRI at baseline than those who did not (all *P <* 0.001). In addition, the proportion of males, Han ethnicity, rural farmers, current smokers, and drinkers was higher in participants who developed hypertension in the process of follow-up. Furthermore, Kaplan‒Meier curves demonstrated that the participants in higher quartiles of AHIs (ABSI or BRI) at baseline were at greater risk of hypertension incidence during the follow-up (log-rank test, P < 0.001; Fig. 1).

**Table 1:** The characteristics of the study population at baseline

Variables	Not developed hypertension	Developed hypertension	*P value*
	N = 7463	N = 4511	
**Age (years)**	37.95 ± 13.27	45.34 ± 13.56	< 0.001
**Height (cm)**	161.00 ± 8.17	160.76 ± 8.49	0.423
**Weight (kg)**	57.23 ± 9.99	59.17 ± 10.26	< 0.001
**HC (cm)**	90.90 ± 7.62	92.37 ± 7.91	< 0.001
**WC (cm)**	76.43 ± 9.23	79.17 ± 9.47	< 0.001
**SBP (mmHg)**	111.38 ± 11.44	115.97 ± 11.33	< 0.001
**DBP (mmHg)**	72.81 ± 8.05	75.52 ± 7.54	< 0.001
**ABSI**	0.077 ± 0.006	0.078 ± 0.006	< 0.001
**BRI**	2.96 ± 1.03	3.30 ± 1.12	< 0.001
**Gender**			< 0.001
Male	3193 (42.78%)	2291 (50.79%)	
Female	4270 (57.2%)	2220 (49.2%)	
**Ethnicity**			0.025
Han	6598 (88.41%)	4048 (89.74%)	
non-Hans	865 (11.59%)	463 (10.26%)	
**Residence**			< 0.001
Urban	2995 (40.13%)	1423 (31.55%)	
Rural	4468 (59.87%)	3088 (68.45%)	
**Current smoke**			< 0.001
No	5449 (73.01%)	2964 (65.71%)	
Yes	2014 (26.99%)	1547 (34.29%)	
**Drinking**			< 0.001
No	5054 (67.72%)	2761 (61.21%)	
Yes	2409 (32.28%)	1750 (38.79%)	

HC: hip circumstance, WC: waist circumstance, SBP: systolic blood pressure, DBP: diastolic blood pressure, ABSI: a body shape index, BRI: body round index

### Univariate analysis for the incidence of hypertension in the whole cohort

In the univariate analysis (Table 2), the criteria associated with the hypertension occurrence were age (HR = 1.04, 95%CI:1.04–1.05)), sex (female vs. male, HR = 0.82, 95%CI:0.77–0.87), race (non-Han vs. Han, HR = 0.76, 95%CI = 0.69–0.84), smoking (HR = 1.15, 95%CI:1.08–1.23), drinking (HR = 1.19, 95%CI:1.13–1.27), SBP (HR = 1.04, 95%:1.04–1.05), DBP (HR = 1.05, 95%CI:1.05–1.06), ABSI z score (HR = 1.25, 95%CI:1.22–1.29), and BRI z score (HR = 1.48, 95%CI = 1.44–1.52). In addition, the higher quartiles of ABSI and BRI were also correlated with the incidence of hypertension.

**Table 2:** Univariate analysis for incidence of hypertension in the whole cohort

Variables	HR (95%CI)	*P value*
Age	1.04 (1.04, 1.05)	*< 0.001*
Sex = female	0.82 (0.77, 0.87)	*<0.001*
Race = non-Han	0.76 (0.69, 0.84)	*< 0.001*
Urban residence	0.95 (0.89, 1.01)	*0.089*
Smoking	1.15 (1.08, 1.23)	*<0.001*
Drinking	1.19 (1.13, 1.27)	*< 0.001*
SBP (mmHg)	1.04 (1.04, 1.05)	*<0.001*
DBP (mmHg)	1.05 (1.05, 1.06)	*<0.001*
**ABSI z score**	1.25 (1.22, 1.29)	*<0.001*
ABSI Q1	*reference*	
ABSI Q2	1.13 (1.26–1.52)	*0.007*
ABSI Q3	1.47 (1.35–1.60)	*<0.001*
ABSI Q4	1.89 (1.73–2.95)	*<0.001*
**BRI z score**	1.48 (1.44, 1.52)	*<0.001*
BRI Q1	*reference*	
BRI Q2	1.39 (1.26–1.52)	*<0.001*
BRI Q3	1.94 (1.77–2.12)	*<0.001*
BRI Q4	2.98 (2.73–3.25)	*<0.001*

SBP: systolic blood pressure, DBP: diastolic blood pressure, ABSI: a body shape index, BRI: body round index, HR: hazard ratio, CI: confidence interval

### The association between AHIs and the incidence of hypertension

After adjusting for age, sex, race, residence, smoking, drinking, SBP, and DBP (model 2), the Cox proportional hazard model analysis (Table 3) showed that BRI (Q2 vs. Q1:HR = 1.24, 95%CI:1.13–1.36; Q3 vs. Q1:HR = 1.55, 95%CI:1.42–1.69; Q4 vs. Q1:HR = 1.99, 95%CI:1.82–2.19) were significantly associated with increased risk of hypertension in the whole cohort but were relatively weak for ABSI quartiles (*P* for trend = 0.387). In addition, ABSI z score and BRI z score were positively associated with an increased incidence of hypertension in the whole cohort. The risk of hypertension increased by 8.0% (95% CI: 1.04–1.11) and 27% (95% CI: 1.23–1.30) for every 1 z score increase in ABSI and BRI, respectively.

**Table 3:** The association of anthropometric indices with incidence of hypertension by Cox regression

Variables	Q1	Q2	Q3	Q4	*P for trend*	Z Score
	HR (95%CI)					HR (95%CI)
**ABSI**						
Model 1	reference	1.01 (0.93, 1.10)	1.16 (1.06, 1.26)	1.27 (1.16, 1.38)	*0.977*	1.09 (1.06, 1.13)
Model 2	reference	1.01 (0.93, 1.10)	1.15 (1.06, 1.26)	1.22 (1.12, 1.33)	*0.387*	1.08 (1.04, 1.11)
**BRI**						
Model 1	reference	1.28 (1.17, 1.40)	1.64 (1.50, 1.79)	2.32 (2.12, 2.54)	*< 0.001*	1.35 (1.31, 1.39)
Model 2	reference	1.24 (1.13, 1.36)	1.55 (1.42, 1.69)	1.99 (1.82, 2.19)	*< 0.001*	1.27 (1.23, 1.30)

ABSI: a body shape index, BRI: body round index, HR: hazard ratio, CI: confidence interval;

Model 1: adjusted for age, sex, race, and residence; Model 2: adjusted for age, sex, race, residence, smoking, drinking, systolic blood pressure, and diastolic blood pressure. The anthropometric measures were converted to a z score using the equation (X-X_mean_)/X_S_

### Stratified analysis on AHIs and the incidence of hypertension

To explore the effect of other variables on the relationship between AHIs and the incidence of hypertension, stratified analysis and interaction tests were conducted. The results (Table 4) showed a greater risk of new-onset hypertension in those < 40 years old (HR = 1.43, 95% CI: 1.35–1.50) for each z score increase in BRI and a higher incidence of hypertension in participants who were drinkers (HR = 1.10, 95% CI: 1.04–1.14) for each z score increase in ABSI. However, the other factors, including sex, race, residence, smoking, and blood pressure levels, did not impact the relationship between the AHIs and the incidence of hypertension.

**Table 4:** Stratified analysis of anthropometric indices and incidence of hypertension

subgroup	sample size	HR (95%CI)	P interaction	HR (95%CI)	P interaction
		ABSI z score		BRI z score	
**Baseline age**					
< 40	6176	1.09 (1.04, 1.15)	0.285	1.43 (1.35, 1.50)	<0.001
>=40	5798	1.06 (1.02, 1.10)		1.20(1.15, 1.24)	
**Sex**					
Male	5484	1.06 (1.01 1.10)	0.282	1.28 (1.22, 1.34)	0.279
Female	6490	1.08 (1.04, 1.13)		1.25 (1.20, 1.30)	
**Race**					
Hans	10,646	1.08 (1.04, 1.11)	0.953	1.27(1.23, 1.30)	0.795
Non-Hans	1328	1.08 (0.98, 1.19)		1.28(1.17, 1.40)	
**Residence**					
Urban	4418	1.05 (1.00, 1.11)	0.444	1.24 (1.18, 1.30)	0.193
Rural	7556	1.09 (1.05, 1.13)		1.28 (1.24, 1.33)	
**Smoking**					
Yes	8413	1.07 (1.03, 1.11)	0.777	1.25 (1.20, 1.29)	0.104
No	3561	1.08 (1.03, 1.14)		1.32 (1.25, 1.40)	
**Drinking**					
Yes	7815	1.10 (1.05, 1.14)	0.041	1.27(1.23, 1.32)	0.346
No	4159	1.03 (0.98, 1.09)		1.25 (1.19, 1.31)	
**Systolic blood pressure**					
< 120 mmHg	7634	1.07 (1.02, 1.11)	0.927	1.28 (1.22, 1.34)	0.367
>=120 mmHg	4340	1.08 (1.03, 1.12)		1.24 (1.20, 1.30)	
**Diastolic blood pressure**					
< 80 mmHg	8021	1.08 (1.04, 1.03)	0.513	1.29 (1.23, 1.34)	0.182
>=80 mmHg	3953	1.07 (1.02, 1.11)		1.24 (1.19, 1.30)	

ABSI: a body shape index, BRI: body round index, HR: hazard ratio, CI: confidence interval;

The anthropometric measures were converted to z scores using the equation: (X-X_mean_)/X_SD;_

Each stratification adjusted for all the factors (age, sex, race, residence, smoking, drinking, systolic blood pressure, and diastolic blood pressure) except the stratification factor itself

### Additional analysis on AHIs’ discrimination of hypertension incidence

#### Comparison of AHIs for identifying the development of hypertension

**Fig. 2:**
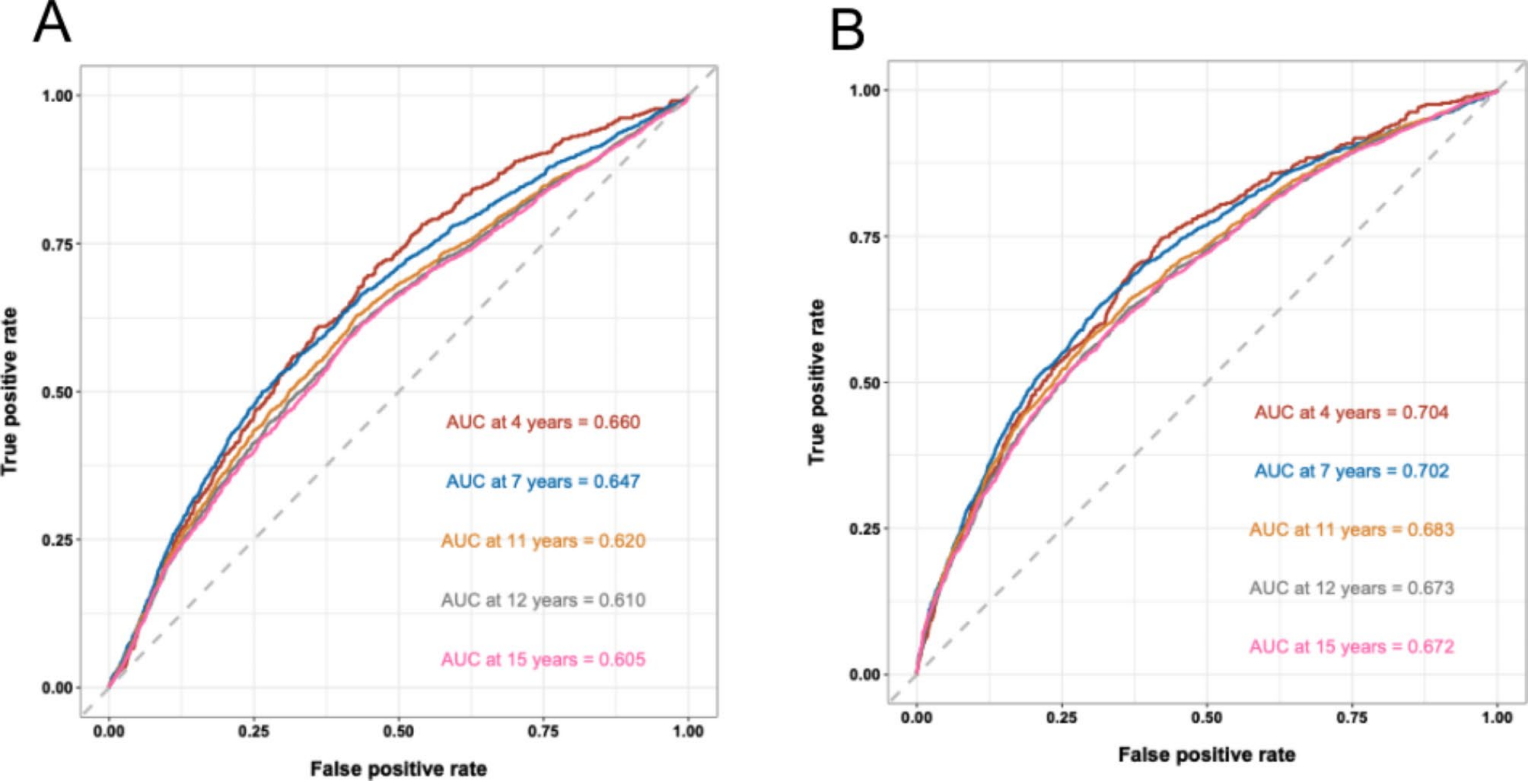
Time-dependent receiver operating characteristics curve analysis for AHIs to discriminate the incidence of hypertension at different time points. A: for ABSI; B: for BRI

**Fig. 3:**
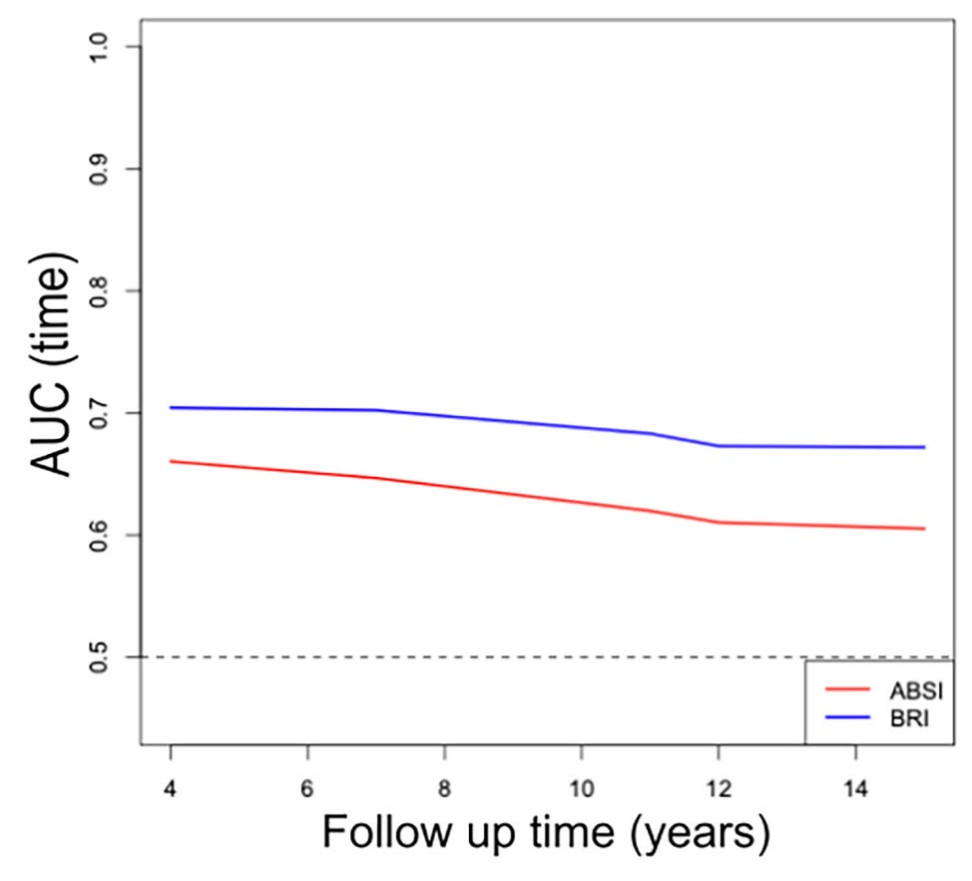
Comparison of the AUC for AHIs to discriminate the incidence of hypertension over time

Time-dependent ROC curve analysis was conducted to analyze the ability of ABSI or BRI to identify new-onset hypertension at different time points. The AUC was 0.660 (95% CI: 0.636–0.685) at 4 years, 0.647 (95% CI: 0.632–0.662) at 7 years, 0.620 (95% CI: 0.606–0.634) at 11 years, 0.610 (95% CI: 0.597–0.624) at 12 years, and 0.605 (95% CI: 0.591–0.620) at 15 years for ABSI. In addition, the AUC was 0.704 (95% CI: 0.680–0.729) at 4 years, 0.702 (95% CI: 0.688–0.717) at 7 years, 0.683 (95% CI: 0.670–0.697) at 11 years, 0.673 (95% CI: 0.660–0.686) at 12 years, and 0.672 (95% CI: 0.659–0.686) at 15 years for BRI. These abovementioned results were shown in Fig. 2. We observed that the AUC for BRI was significantly higher than that of ABSI at any time point we tested (Table 5). Furthermore, the AUC of both indices decreased over time in this study (Fig. 3).

**Table 5:** The AUC and corresponding 95% CI of anthropometric indices for hypertension incidence at different time points

Time	AUC(95%CI)		*P value*	
	ABSI	BRI		
4 years	0.660(0.636–0.685)	0.704(0.680–0.729)		*< 0.001*
7 years	0.647(0.632–0.662)	0.702(0.688–0.717)		*< 0.001*
11 years	0.620(0.606–0.634)	0.683(0.670–0.697)		*< 0.001*
12 years	0.610(0.597–0.624)	0.673(0.660–0.686)		*< 0.001*
15 Years	0.605(0.591–0.620)	0.672(0.659–0.686)		*< 0.001*

ABSI: body shape index, BRI: body round index, HR: hazard ratio, CI: confidence interval;

AUC: area under the curve, CI: confidence interval

#### Preliminary exploration on the predictive value of AHIs for hypertension incidence

We further analyzed the incremental effect of both indices on the predictive value for hypertension incidence at 7 years (the median follow-up time of the present study). The results (Table 6) demonstrated that the addition of BRI improved the differentiation and reclassification of traditional risk factors with a continuous NRI of 0.201 (95% CI: 0.169–0.228) and an IDI of 0.021 (95% CI: 0.015–0.028). However, the addition of ABSI did not improve discrimination, with an IDI of 0.003 (95% CI: -0.001-0.008).

**Table 6:** The value of ABSI or BRI improved the risk stratification of hypertension incidence according to continuous-NRI and IDI

	C-index	IDI		continuous-NRI	
		Est.(95%CI)	*P value*	Est.(95%CI)	*P value*
traditional risk factors	0.737	**Ref.**		**Ref.**	
traditional risk factors + BRI	0.746	0.021 (0.015–0.028)	<0.001	0.201 (0.169–0.228)	<0.001
traditional risk factors + ABSI	0.738	0.003 (-0.001-0.008)	0.169	0.107 (0.063–0.128)	<0.001

Traditional risk factors: age, sex, race, residence, smoking, drinking, SBP, and DBP;

ABSI: a body shape index, BRI: body round index, CI: confidence interval;

IDI: integrated discrimination improvement; NRI: net reclassification index

## Discussion

Our study using prospective data from CHNS demonstrated that increased baseline AHIs were associated with an increased incidence of hypertension over a median of 7.0 years of follow-up in Chinese individuals. Of them, BRI performed better than ABSI to discriminate hypertension incidence at different time points. Furthermore, the addition of BRI had a better predictive value for hypertension incidence than ABSI by analysis of IDI and NRI.

To date, four categories with 42 AHIs have been developed, which have been widely used for risk identification of nutritional status, cardiovascular disease, metabolic disorders, and health management [21]. Most of the AHIs are estimated according to 3D human shapes and can be easily obtained by noninvasive, inexpensive measurements in health checks. The most commonly used parameters include traditional indices, namely, weight, height, body mass index (BMI), WC, HC, and waist-to-hip ratio (WHR). The novel AHIs include the ABSI [8], BRI [9], etc. These AHIs were regarded as a suitable screening tool for the early detection of cardiovascular disease and to reduce the associated medical costs [22].

Traditional AHIs, such as BMI and WHR, are usually used to evaluate overweight/obesity and are correlated with the incidence of hypertension. Hu et al. demonstrated that increasing BMI levels were significantly associated with hypertension incidence in Finnish male and female participants when not adjusted for baseline SBP in Cox regression. However, this correlation weakened after a further adjustment for SBP [23]. However, BMI has some deficiencies because it cannot address visceral fat or fat distribution and cannot differentiate between excess fat and high muscle mass [21]. WHR, an indicator of visceral fat, is calculated by WC divided by HC. It might be inaccurate when BMI exceeds 35 kg/m^2^ [21]. Lee et al. suggested that WHR was correlated with the incidence of hypertension in both sexes of middle-aged Korean people [7]. To date, some studies have compared the ability to identify hypertension using ABSI or BRI by conducting a cross-sectional study [11, 12, 15]. However, only one longitudinal study evaluated the correlation between novel AHIs and the incidence of hypertension and explored their ability to discriminate hypertension incidents in a Korean population [16]. Therefore, the relationship between novel AHIs and the incidence of hypertension in the Chinese population is unclear.

ABSI and BRI are both novel AHIs developed by Krakauer et al. in 2012 and by Thomas et al. in 2013, respectively [8, 9]. ABSI is a parameter based on WC adjusted for weight and height and reflects body shape, abdominal size, and concentration of body volume [21, 24]. ABSI is associated with the onset of diabetes, metabolic syndrome, and carotid atherosclerosis [25–28] and can predict all-cause mortality and cardiovascular death [8, 29]. However, it performed poorly in hypertension prediction. First, Calderón-García et al. demonstrated that the estimated pooled AUC for ABSI (AUC = 0.58, 95% CI: 0.56–0.60) for the discrimination of hypertension was the lowest compared with BRI by conducting a systematic review and meta-analysis that included 13 original studies [15]. Second, Choi et al. also suggested that ABSI showed the lowest discrimination power for hypertension compared to other AHIs in a Korean community-based prospective study [16]. Interestingly, of the two novel parameters we analyzed, ABSI was weak in identifying hypertension incidence at different time points. The poor performance of ABSI for hypertension discrimination can be explained as follows. This formula was initially developed to predict mortality instead of hypertension incidence by using longitudinal data from the National Health and Nutrition Examination Survey (NHANES) 1999–2004 [8]. Furthermore, ABSI was developed by the white, black, and Mexican ethnicities of Americans, which might be not suitable for the Chinese population. BRI is based on height and WC, which reflect body shape, visceral adipose tissue, and body fat. The Spearman correlation coefficient between BRI and WHtR was 0.996 in the whole cohort of the present study, which was consistent with Maessen et al. [5]. In 2018, Choi and colleagues found that WHtR was a better predictor of incident hypertension than BMI and WHR [30]. In contrast to the WHtR, the BRI can not only assess body fat percentage but also supply a more accurate estimation of health status. Choi’s subsequent study further proved that the hypertension discrimination ability for BRI was basically equal to that for WHtR, with the same AUC value and 95% CI [16]. In the present study, we found that BRI was positively correlated with hypertension incidence and had a higher AUC value than ABSI for hypertension discrimination at different time points in the whole cohort. Furthermore, the addition of BRI improved the differentiation and reclassification of the traditional risk factors. On the basis of Choi’s study, we further found a greater risk of new-onset hypertension in age < 40 years old for each z score increase in BRI. Similarly, Zhang et al. also found that age might influence the relationship between AHIs and hypertension by conducting a cross-sectional survey in 8234 Chinese adults [31]. These results suggested that it is crucial to choose a proper index for hypertension screening according to the specific age. However, the age difference in AHIs to discriminate hypertension is uncertain. Hence, more prospective studies should be conducted to verify this phenomenon. However, it is worth noting that the formula of BRI is complex compared with the simplicity of other indicators, such as BMI or WHR, which might limit its use and promotion in clinical practice. Hence, developing an automatic calculator for AHIs on websites or mobile phone applications is necessary at this moment.

This study has several strengths. To the best of our knowledge, this is the first study to analyze the relationship between novel AHIs and the incidence of hypertension and compare their ability to discriminate hypertension incidence in the Chinese population. Second, the data in the present study were obtained from a large-scale, long-term, multiprovincial, and population-based prospective cohort. This might supply relatively accurate and comprehensive evidence on the relationship between novel AHIs and the incidence of hypertension. However, some limitations of this study should be stated. First, only Chinese individuals were studied. Therefore, our findings may not be generalizable to other ethnic populations. Second, the data on family history of hypertension and parameters of blood biochemical examination were not collected in the early stage of CHNS. Hence, these potential confounders could not be adjusted. Third, the original question for antihypertensive drugs in the CHNS was as follows: “Are you currently taking antihypertensive drugs: 0 for no and 1 for yes”. Therefore, the antihypertensive medication status was defined by participants’ self-report and investigators’ specialized judgment in this investigation. Owing to this situation and not using the anatomical therapeutic chemical classification codes, errors might be caused because of subjects’ recall bias and different comprehensive understanding abilities. In addition, the nutrients and details of diet intake were not corrected in this study.

## Conclusion

Increased ABSI and BRI were associated with an increased risk of hypertension in Chinese individuals. BRI performed better than ABSI in identifying the new onset of hypertension, and the discrimination ability of both indices decreased over time.

## Data Availability

The datasets generated during and/or analysed during the current study are available from the corresponding author on reasonable request.

## List of abbreviations

AHIs: anthropometric indices
BRI: body roundness index
ABSI: body shape index
BMI: body mass index
WHR: waist-to-hip ratio
CHNS: China Health and Nutrition Survey
ROC: receiver-operating characteristic
AUC: area under the curve
WC: waist circumference
HC: hip circumference
SBP: systolic blood pressure:DBP:diastolic blood pressure
SD: standard deviation
HR: hazard ratio
CI: confidence interval
IDI: integrated discrimination improvement
NRI: net reclassification index.

## References

[1] Brouwers S, Sudano I, Kokubo Y (2021). Arterial hypertension. Lancet.

[2] Wang Z, Chen Z, Zhang L (2018). Status of hypertension in China: results from the China Hypertension Survey, 2012–2015. Circulation.

[3] Zhou M, Wang H, Zeng X (2019). Mortality, morbidity, and risk factors in China and its provinces, 1990–2017: a systematic analysis for the global burden of Disease Study 2017. Lancet.

[4] Wang Z, Hao G, Wang X (2019). Clinical outcomes and economic impact of the 2017 ACC/AHA guidelines on hypertension in China. J Clin Hypertens (Greenwich).

[5] Maessen MF, Eijsvogels TM, Verheggen RJ (2014). Entering a new era of body indices: the feasibility of a body shape index and body roundness index to identify cardiovascular health status. PLoS ONE.

[6] Moosaie F, Fatemi Abhari SM, Deravi N (2021). Waist-To-Height ratio is a more Accurate Tool for Predicting Hypertension Than Waist-To-Hip circumference and BMI in patients with type 2 diabetes: a prospective study. Front Public Health.

[7] Lee JW, Lim NK, Baek TH (2015). Anthropometric indices as predictors of hypertension among men and women aged 40–69 years in the korean population: the Korean Genome and Epidemiology Study. BMC Public Health.

[8] Krakauer NY, Krakauer JC (2012). A new body shape index predicts mortality hazard independently of body mass index. PLoS ONE.

[9] Thomas DM, Bredlau C, Bosy-Westphal A (2013). Relationships between body roundness with body fat and visceral adipose tissue emerging from a new geometrical model. Obes (Silver Spring).

[10] Rico-Martin S, Calderon-Garcia JF, Sanchez-Rey P (2020). Effectiveness of body roundness index in predicting metabolic syndrome: a systematic review and meta-analysis. Obes Rev.

[11] Chang Y, Guo X, Guo L (2016). The feasibility of two new anthropometric indices to identify hypertension in rural China: a cross-sectional study. Med (Baltim).

[12] Baveicy K, Mostafaei S, Darbandi M (2020). Predicting Metabolic Syndrome by Visceral Adiposity Index, Body Roundness Index and a body shape index in adults: a cross-sectional study from the iranian RaNCD Cohort Data. Diabetes Metab Syndr Obes.

[13] Stefanescu A, Revilla L, Lopez T (2020). Using a body shape index (ABSI) and body roundness index (BRI) to predict risk of metabolic syndrome in peruvian adults. J Int Med Res.

[14] Gluszek S, Ciesla E, Gluszek-Osuch M (2020). Anthropometric indices and cut-off points in the diagnosis of metabolic disorders. PLoS ONE.

[15] Calderon-Garcia JF, Roncero-Martin R, Rico-Martin S et al. Effectiveness of Body Roundness Index (BRI) and a Body Shape Index (ABSI) in Predicting Hypertension: A Systematic Review and Meta-Analysis of Observational Studies. Int J Environ Res Public Health, 2021. 18(21). 10.3390/ijerph182111607 PMC8582804 34770120

[16] Choi JR, Ahn SV, Kim JY (2018). Comparison of various anthropometric indices for the identification of a predictor of incident hypertension: the ARIRANG study. J Hum Hypertens.

[17] Popkin BM, Du S, Zhai F (2010). Cohort Profile: the China Health and Nutrition Survey–monitoring and understanding socio-economic and health change in China, 1989–2011. Int J Epidemiol.

[18] Guo J, Zhu YC, Chen YP (2015). The dynamics of hypertension prevalence, awareness, treatment, control and associated factors in chinese adults: results from CHNS 1991–2011. J Hypertens.

[19] Chobanian AV, Bakris GL, Black HR (2003). The Seventh Report of the Joint National Committee on Prevention, detection, evaluation, and treatment of high blood pressure: the JNC 7 report. JAMA.

[20] Chandra A, Neeland IJ, Berry JD (2014). The relationship of body mass and fat distribution with incident hypertension: observations from the Dallas Heart Study. J Am Coll Cardiol.

[21] Piqueras P, Ballester A, Dura-Gil JV (2021). Anthropometric indicators as a Tool for diagnosis of obesity and other Health risk factors: a Literature Review. Front Psychol.

[22] Darbandi M, Pasdar Y, Moradi S (2020). Discriminatory capacity of Anthropometric Indices for Cardiovascular Disease in adults: a systematic review and Meta-analysis. Prev Chronic Dis.

[23] Hu G, Barengo NC, Tuomilehto J (2004). Relationship of physical activity and body mass index to the risk of hypertension: a prospective study in Finland. Hypertension.

[24] Krakauer NY, Krakauer JC (2018). Untangling Waist circumference and hip circumference from body Mass Index with a body shape index, hip index, and Anthropometric Risk Indicator. Metab Syndr Relat Disord.

[25] He S, Chen X (2013). Could the new body shape index predict the new onset of diabetes mellitus in the chinese population?. PLoS ONE.

[26] Lee DY, Lee MY, Sung KC (2018). Prediction of mortality with a body shape index in Young Asians: comparison with body Mass Index and Waist circumference. Obes (Silver Spring).

[27] Bertoli S, Leone A, Krakauer NY (2017). Association of body shape index (ABSI) with cardio-metabolic risk factors: a cross-sectional study of 6081 caucasian adults. PLoS ONE.

[28] Geraci G, Zammuto M, Gaetani R (2019). Relationship of a body shape index and body roundness index with carotid atherosclerosis in arterial hypertension. Nutr Metab Cardiovasc Dis.

[29] Song X, Jousilahti P, Stehouwer CD (2015). Cardiovascular and all-cause mortality in relation to various anthropometric measures of obesity in Europeans. Nutr Metab Cardiovasc Dis.

[30] Choi JR, Koh SB, Choi E (2018). Waist-to-height ratio index for predicting incidences of hypertension: the ARIRANG study. BMC Public Health.

[31] Zhang B, Fan Y, Wang Y (2021). Comparison of bioelectrical body and visceral fat indices with anthropometric measures and optimal cutoffs in relation to hypertension by age and gender among chinese adults. BMC Cardiovasc Disord.

